# Synergistic Antibacterial Effect of *Eisenia bicyclis* Extracts in Combination with Antibiotics against Fish Pathogenic Bacteria

**DOI:** 10.4014/jmb.2406.06027

**Published:** 2024-07-22

**Authors:** Raul Joao Lourenco Mascarenha, Du-Min Jo, Yoon-Ah Sim, Do-Hyung Kim, Young-Mog Kim

**Affiliations:** 1 KOICA-PKNU International Graduate Program of Fisheries Science, Pukyong National University, Busan 48513, Republic of Korea; 2 National Marine Biodiversity Institute of Korea, Seochun 33662, Republic of Korea; 3 Department of Food Science and Technology, Pukyong National University, Busan 48513, Republic of Korea; 4 Department of Aquatic Life Medicine, Pukyong National University, Busan 48513, Republic of Korea

**Keywords:** *Eisenia bicyclis*, antibiotic resistance, fish pathogenic bacteria, synergy

## Abstract

The aquaculture industry faces significant challenges due to bacterial infections caused by *Edwardsiella tarda*, *Photobacterium damselae*, and *Vibrio harveyi*. The extensive use of traditional antibiotics, has resulted in widespread antibiotic resistance. This study aimed to investigate the antibacterial potential of the brown seaweed *Eisenia bicyclis*, particularly its synergistic effects with antibiotics against these fish pathogenic bacteria. *E. bicyclis* were processed to obtain methanolic extracts and fractionated using different polar solvents. The antibacterial activities of these extracts and fractions were assessed through disc diffusion and minimum inhibitory concentration (MIC) assays. The study further evaluated the antibiotic susceptibility of the bacterial strains and the synergistic effects of the extracts combined with erythromycin and oxyteteracycline using the fractional inhibitory concentration index. Results showed that the ethyl acetate (EtOAc) fraction of *E. bicyclis* methanolic extract exhibited the highest antibacterial activity. The combination of the EtOAc fraction with erythromycin significantly enhanced its antibacterial efficacy against the tested strains. This synergistic effect was indicated by a notable reduction in MIC values, demonstrating the potential of *E. bicyclis* to enhance the effectiveness of traditional antibiotics. The findings suggest that *E. bicyclis* extracts, particularly the EtOAc fraction, could serve as a potent natural resource to counteract antibiotic resistance in aquaculture.

## Introduction

Edwarsiella tarda, *Photobacterium damselae*, and *Vibrio harveyi* are Gram-negative, facultatively anaerobic bacteria known to be significant fish pathogens [1]. *E. tarda* is responsible for severe diseases in both freshwater and marine fish, with reported mortality rates of up to 90% [2]. *P. damselae* has emerged as a major disease-causing agent in sea bream (*Sparus aurata*) and is a primary pathogen for various marine animals, including crustaceans, mollusks, and cetaceans [3]. Recently, it has also been recognized as a major pathogen in newly farmed aquaculture fish species [4]. Similarly, *V. harveyi* is a pathogen that causes severe diseases in penaeid shrimp and fish, with shrimp larvae experiencing mortality rates of up to 100% [5, 6]. Despite the long-term use of traditional antibiotics to manage disease outbreaks in aquaculture, these treatments have proven increasingly ineffective. The widespread and prolonged application of antibiotics like tetracycline and its derivative oxytetracycline (OTC), as well as erythromycin (ERY), has led to significant resistance issues [7]. The high stocking densities typical of modern aquaculture systems exacerbate this problem by causing excessive stress in fish, which increases their susceptibility to bacterial infections [8, 9]. Bacterial pathogens in aquatic environments adapt readily through genome amplification and horizontal gene transfer, contributing to antibiotic resistance and consequent treatment failures [10]. These genetic adaptations result in structural changes in the target cells, rendering antibiotics less effective and leading to persistent disease outbreaks [11]. This resistance not only affects the aquaculture industry economically but also poses health risks to farm workers and consumers [12, 13]. A study by the UK government predicts that by 2050, 10 million people could die annually from infections caused by multidrug-resistant bacteria [14]. Given the growing demand for fish as a primary source of animal protein, the development of antibiotic resistance in aquaculture is a global concern [15]. This urgent issue has driven the search for alternative treatments with strong antibiotic properties. Recent research has increasingly focused on seaweed due to its potential as a sustainable and effective alternative to synthetic drugs [16]. *Eisenia bicyclis*, a brown seaweed, contains polysaccharides that exhibit various bioactivities, including antioxidant, anti-Alzheimer, anticancer, anti-atherosclerosis, anti-inflammatory, anti-allergic, and anticoagulant properties[17-20]. Notably, this seaweed has been shown to contain active metabolites with significant antibiotic properties and the ability to enhance the efficacy of existing drugs against resistant bacterial strains [21].

This study aims to evaluate the antibiotic potential of *E. bicyclis* and investigate its synergistic effects with erythromycin (ERY) and oxytetracycline (OTC) against selected fish pathogenic bacteria. By exploring the antibacterial properties of this brown seaweed, we hope to identify effective alternatives to traditional antibiotics and contribute to the development of sustainable disease management strategies in aquaculture.

## Materials and Methods

### Plant Materials

Fresh samples of *Eisenia bicyclis* were directly purchased from Ulleung Trading Co. (Republic of Korea). The plant samples were thoroughly washed with tap water and allowed to shade-dry for one week. Subsequently, the samples were dried in a vacuum oven (ThermoStable OV-30; DAIHAN Scientific Co. Ltd., Republic of Korea) at 60°C for 24 h. The dried *E. bicyclis* was ground into powder using a grinder.

### Preparation of Extract and Fractions

The dried *E. bicyclis* powder was extracted three times with 70% methanol at a ratio of 1:3 (w/v) at 70°C for 3 h with continuous stirring at 300 rpm. The obtained extract was concentrated using a rotary evaporator (EYELA Co., Japan) under vacuum at 40°C, yielding 125 g of crude methanol extract. This extract was resuspended in 1 L of 10% methanol and subsequently fractionated in sequence using *n*-hexane (Hexane; 270504, Sigma-Aldrich, USA), dichloromethane (DCM; 650463, Sigma-Aldrich), ethyl acetate (EtOAc; 34858, Sigma-Aldrich), and *n*-butanol (BuOH; 34867, Sigma-Aldrich) at a ratio of 1:1 (w/v).

### Bacterial Strains and Culture Conditions

The bacterial strains *E. tarda*, *P. damselae*, and *V. harveyi* were obtained from the Fish Disease Prevention Lab, Department of Aquatic Life Medicine, Pukyong National University (Republic of Korea). These strains were previously isolated from fish sources. The bacteria were inoculated and cultured anaerobically in tryptic soy broth (TSB; 211825, Difco, USA) supplemented with 1% NaCl.

### Disc Diffusion Assay

The disc diffusion assay was performed to determine the antibacterial activity of the extracts, following the guidelines of the Clinical and Laboratory Standards Institute [22]. *E. tarda*, *P. damselae*, and *V. harveyi* were adjusted to a density of 10^8^ CFU/ml (0.5 on the McFarland scale) and spread on Mueller-Hinton agar (MHA; 275730, Difco) plates. Sterile paper discs (6 mm in diameter) were loaded with 1 mg/disc and 5 mg/disc of the crude methanolic extract and its fractions (Hexane, DCM, EtOAc, BuOH, and water) and placed onto the MHA plates. The plates were incubated at 35°C for 24 h. Erythromycin (ERY; E5389, Sigma-Aldrich) and oxytetracycline (OTC; 1491004, Sigma-Aldrich) were used as controls at concentrations of 5 mg/disc and 30 mg/disc, respectively. The diameter of the inhibition zones (mm) was measured to evaluate antibacterial activity. All tests were performed in triplicate.

### Minimum Inhibitory Concentration (MIC) Assay

The MIC was determined using a standard two-fold serial dilution method in 96-well microtiter plates [22]. Serial dilutions of the extract and fractions, starting from 1,024 μg/ml, were prepared in 96-well microtiter plates containing 10^4^ CFU/ml of bacterial culture and incubated at 35°C for 24 h. Dimethyl sulfoxide (DMSO) and bacterial strains were used as controls. Optical density (OD_600 nm_) of the samples was measured using a Synergy HTX Multi-Mode Microplate Reader (Biotek, Republic of Korea). All assays were performed in triplicate.

### Antibiotic Susceptibility Test

Antibiotic susceptibility test was performed to evaluate the response of bacterial strains to ERY and OTC, using the Kirby-Bauer disk diffusion method [22]. Bacterial strains were inoculated on MHA plates at a concentration of 10^5^ CFU/ml, and discs loaded with 5 mg/disc and 30 mg/disc of ERY and OTC, respectively, were placed on the plates. The plates were incubated at 35°C for 24 h, and the diameter of the inhibition zones was measured.

### Synergy of Fractional Inhibitory Concentration (FIC)

The FIC index was used to assess the synergistic effects of *E. bicyclis* methanolic extract and its fractions in combination with ERY and OTC against *E. tarda*, *P. damselae*, and *V. harveyi*. Synergy was indicated by an FIC index of ≤ 0.5, additive effects by > 0.5 to ≤ 1, independent effects by > 1 to ≤ 2, and antagonistic effects by > 2 [23]. The FIC was calculated using the formula:

FIC_A_ = C_A_/MIC_A_

FIC_B_ = C_B_/MIC_B_

FIC Index = FIC_A_ + FIC_B_

where MIC_A_ and MIC_B_ are the MIC of each compound A and B, respectively, and C_A_ and C_B_ are the MIC of the compounds in combination [24].

### Statistical Analysis

All experiments were performed in triplicate, and the data were averaged. Standard deviations were calculated. Multiple comparisons were evaluated by two-way ANOVA using IBM SPSS Statistics Version 25. Significant differences between means were determined using Tukey's test, with *p* < 0.05 considered significant.

## Results and Discussion

### Antibacterial Activity of *E. bicyclis* against Fish Pathogenic Bacteria by Disc Diffusion Assay

Solvent fractionation of crude *E. bicyclis* seaweed extract (300 g) yielded six different soluble fractions: Hexane (1.5 g), DCM (0.17 g), EtOAc (9.09 g), BuOH (7.61 g), and H_2_O. The antibacterial efficacy of these fractions was determined by measuring the inhibition zone diameter in a disc diffusion assay, as shown in Table 1. Increasing the concentration from 1 mg/disc to 5 mg/disc significantly increased the size of the inhibition zone. The BuOH fraction's inhibition zone increased from 7 to 11.3 mm for *E. tarda* EET34 and from 7.6 to 14.3 mm for *V. harveyi* FRHW1KA. Particularly, the EtOAc and BuOH fractions exhibited significant antibacterial activity against all tested bacterial strains. Among all extracts and fractions of *E. bicyclis*, the EtOAc fraction showed the highest antibacterial effect against *V. harveyi* AP9L, with inhibition diameters of 11.3 mm and 19.6 mm at 1 mg and 5 mg/disc, respectively. The methanolic extract and water fraction showed no antibacterial activity at 1 mg/disc and only minor activity at 5 mg/disc.

### Antibacterial Activity of *E. bicyclis* against Fish Pathogenic Bacteria by MIC Assay

The MIC values for nine fish pathogenic bacteria using the methanolic extract and each fraction are shown in Table 2. The results quantitatively demonstrated the inhibitory activities of *E. bicyclis* seaweed extract and its fractions. The MIC values varied depending on the type of extract and bacterial strains, ranging from 128 to 1,024 μg/ml. The EtOAc fraction exhibited the strongest antibacterial activities against all strains, with MIC values ranging from 128 to 256 μg/ml. Additionally, *E. tarda* EET53 and *E. tarda* EET54 showed antibacterial effects even at relatively low concentrations in the extract and all fractions.

### Antibiotic Susceptibility in Fish Pathogenic Bacteria

Tetracycline and its derivative oxytetracycline (OTC) are among the most widely used antibiotics in aquaculture, while erythromycin (ERY) ranks sixth among top medications for treating fish diseases [25]. As shown in Table 3, the tested bacteria have developed resistance to these antibiotics. Long-term use of this family of drugs often leads to a loss of bactericidal potency [26]. The application of ERY, a 50S ribosome inhibitor, and OTC, a 30S ribosome inhibitor, against fish pathogenic bacteria resulted in varying inhibition zone sizes. ERY produced inhibition zones of 11 mm and 29 mm against *P. damselae* FP4137 and *E. tarda* EET53, respectively. In contrast, OTC produced inhibition zones of 9.6 mm and 41.6 mm against *E. tarda* EET34 and *V. harveyi* RFHW3KA, respectively. However, strains such as *E. tarda* EET34, *P. damselae* FP2137, *P. damselae* FP2261, and *V. harveyi* RFHW1KA exhibited resistance to OTC, with inhibition zones ranging from 9.6 to 20.3 mm. This resistance is likely due to mutations in rRNA, which prevent OTC from blocking aminoacyl-tRNA access to the ribosome [27]. The main mechanism of resistance involves differences in bacterial cell wall structure and outer membrane composition [28, 29]. As a result, *E. tarda* EET34 displayed intermediate susceptibility to OTC. Overall, these findings highlight the complex nature of antibiotic resistance in fish pathogenic bacteria. The resistance mechanisms, primarily mutations in rRNA and changes in cell wall structure, underscore the need for ongoing surveillance and development of new strategies to manage bacterial infections in aquaculture.

### ERY and OTC Susceptibility by MIC Assay

The strains *P. damselae* FP4137, *V. harveyi* RFHW9L, and *V. harveyi* RFHW1KA showed resistance to ERY. All *E. tarda* strains, along with *P. damselae* FP2137 and FP2261, and *V. harveyi* RFHW1KA, exhibited resistance to OTC (Table 3). This resistance suggests that these bacteria have developed mechanisms to counteract the bactericidal effects and generate reactive oxygen species in response [30]. *E. tarda* EET34 showed intermediate susceptibility to ERY. Table 4 shows that most *E. tarda* strains and *P. damselae* FP2137 and FP2261 strains were susceptible to ERY. In contrast, *P. damselae* FP4137, *V. harveyi* RFHW9L, and *V. harveyi* RFHW3KA were highly susceptible to OTC. The selected bacterial strains *E. tarda* EET34, *P. damselae* FP4137, *V. harveyi* RFHW9L, and *V. harveyi* RFHW1KA did not show high MIC values for ERY, indicating no acquired resistance. However, the synergistic effect of ERY with the EtOAc fraction significantly enhanced antibacterial activity, reducing MIC values for *P. damselae* FP4137 and *V. harveyi* RFHW9L from 256 μg/ml to 64 μg/ml. According to Table 5, selected strains of *E. tarda* EET53, *E. tarda* EET54, *P. damselae* FP2137, *P. damselae* FP2261, and *V. harveyi* strains did not exhibit high MIC values for OTC, indicating no resistance. However, the combination of OTC with the EtOAc fraction resulted in antagonism. The study findings align with previous reports of synergism between bioactive molecules in the EtOAc fraction and ERY and interactions between antibiotics [31, 32]. This combination could positively impact aquaculture, microbiology, and pharmacology [33, 34]. The antagonistic effect of EtOAc and OTC suggests that their bioactive compounds are inherently divergent and cannot jointly disrupt bacterial cell pathways at the tested concentrations [35, 36].

## Conclusion

This study demonstrates the significant potential of *Eisenia bicyclis* as an alternative therapeutic resource to address the pervasive issue of multidrug resistance in aquaculture. These findings show that the combination of traditional antibiotics with natural antibacterial agents can effectively enhance antibacterial activity against pathogenic bacteria. *E. bicyclis*, rich in phenolic compounds, was utilized to augment the antibacterial properties of methanolic extracts through fractionation with various polar solvents. Notably, the ethyl acetate (EtOAc) fraction of *E. bicyclis* methanolic extract significantly enhanced the antibacterial efficacy of erythromycin (ERY) against selected fish pathogenic bacterial strains. Such studies should also consider the potential for bacteria to develop resistance to *E. bicyclis* extracts over time, similar to antibiotic resistance. Additionally, analyzing the resistance patterns of bacteria over prolonged use of *E. bicyclis* extracts is crucial. Given the extensive use of ERY and oxytetracycline (OTC) in the aquaculture industry, coupled with the documented resistance of some bacterial strains to these antibiotics, our findings are particularly relevant. This result suggests that incorporating *E. bicyclis* extracts with ERY could serve as a potent strategy to counteract antibiotic resistance in aquaculture. To build on these promising results, further research is needed to thoroughly evaluate the synergistic effects of these combinations and their influence on the biology and ecology of the bacterial strains. Such studies could lead to the development of more effective and sustainable disease management strategies, ultimately benefiting the aquaculture industry. Ultimately, this research could lead to the development of more effective and sustainable disease management strategies, benefiting the aquaculture industry while mitigating the risk of resistance development and ensuring environmental sustainability.

## Figures and Tables

**Table 1:** Disc diffusion assay of *Eisenia bicyclis* methanolic extract and its solvent-soluble fractionations.

Bacterial strain	Concentration	Zone of inhibition (mm)					
		MeOH	Hexane	DCM	EtOAc	BuOH	H_2_O
*E. tarda*	1 mg/disc	-	7.0 ± 0.3^b^*	7.0 ± 0.2^b^	8.0 ± 0.2^a^	7.0 ± 0.5^b^	-
EET34	5 mg/disc	7.0 ± 0.2^d^	8.6 ± 0.3^b^	8.0 ± 0.3^c^	11.0 ± 0.2^a^	11.3 ± 0.5^a^	7.0 ± 0.2^d^
*E. tarda*	1 mg/disc	-	-	7.0 ± 0.2^a^	7.3 ± 0.5^a^	7.0 ± 0.3^a^	-
EET53	5 mg/disc	-	7.0 ± 0.5^d^	8.0 ± 0.2^c^	9.6 ± 0.5^b^	12.0 ± 0.4^a^	7.0 ± 0.3^d^
*E. tarda*	1 mg/disc	-	-	-	7.0 ± 0.4^a^	7.0 ± 0.4^a^	-
EET54	5 mg/disc	7.0 ± 0.3^d^	7.6 ± 0.3^c^	8.0 ± 0.3^c^	10.0 ± 0.4^b^	12.0 ± 0.2^a^	-
*P. damselae*	1 mg/disc	-	-	-	8.0 ± 0.2^a^	8.0 ± 0.5^a^	-
FP2137	5 mg/disc	10.0 ± 0.3^c^	7.0 ± 0.2^e^	-	14.0 ± 0.2^a^	12.0 ± 0.6^b^	9.0 ± 0.2^d^
*P. damselae*	1 mg/disc	-	-	-	9.0 ± 0.3^a^	8.0 ± 0.2^b^	-
FP2261	5 mg/disc	10.0 ± 0.4^c^	9.0 ± 0.2^d^	8.0 ± 0.3^e^	14.0 ± 0.2^a^	13.0 ± 0.7^b^	8.3 ± 0.2^e^
*P. damselae*	1 mg/disc	-	-	-	-	8.0 ± 0.3	-
FP4137	5 mg/disc	7.0 ± 0.2^b^	-	-	9.6 ± 0.2^a^	7.3 ± 0.3^b^	-
*V. harveyi*	1 mg/disc	7.0 ± 0.5^c^	7.0 ± 0.1^c^	-	11.3 ± 0.2^a^	9.0 ± 0.5^b^	-
AP9L	5 mg/disc	12.6 ± 0.5^c^	9.0 ± 0.3^de^	9.6 ± 0.3^d^	19.6 ± 0.3^a^	16.0 ± 0.4^b^	8.6 ± 0.3^e^
*V. harveyi*	1 mg/disc	-	-	-	8.0 ± 0.3^a^	7.6 ± 0.5^a^	-
FRHW1KA	5 mg/disc	10.0 ± 0.2^c^	7.0 ± 0.3^e^	7.0 ± 0.3^e^	12.0 ± 0.3^b^	14.3 ± 0.8^a^	8.0 ± 0.3^d^
*V. harveyi*	1 mg/disc	-	-	-	8.0 ± 0.4^b^	10.0 ± 0.2^a^	-
RFHW3KA	5 mg/disc	8.6 ± 0.2^b^	7.6 ± 0.3^c^	7.0 ± 0.4^c^	13.6 ± 0.2^a^	14.0 ± 0.6^a^	7.6 ± 0.3^c^

*E. tarda*, *Edwardsiela tarda*. *P. damselae*, *Photobacterium demsielae*. *V. harveyi*, *Vibrio harveyi*. BuOH, *n*-butanol soluble extract.

DCM, dichloromethane soluble extract. EtOAc, ethyl acetate soluble extract. Hexane, *n*-hexane soluble extract. H_2_O, distilled water soluble extract. Data are the averages of duplicate experiments. -, no detected antibacterial activity. *Values sharing the same letters within each row are not significantly different at *p* < 0.05.

**Table 2:** Minimum inhibitory concentration (MIC) of *Eisenia bicyclis* methanolic extract and its solventsoluble fractionations.

Bacterial Strain	MIC (μg/ml)					
	MeOH	Hexane	DCM	EtOAc	BuOH	DW
*E. tarda* EET34	256	512	512	128	256	512
*E. tarda* EET53	256	512	256	128	256	512
*E. tarda* EET54	256	512	256	128	256	512
*P. damselae* FP2137	256	512	512	256	256	512
*P. damselae* FP2261	256	512	512	256	256	512
*P. damselae* FP4137	512	1024	1024	128	1024	1024
*V. harveyi* AP9L	512	128	512	128	256	512
*V. harveyi* FRHW1KA	256	512	512	128	256	1024
*V. harveyi* RFHW3KA	256	512	512	256	256	1024

*E. tarda*, *Edwardsiela tarda*. *P. damselae*, *Photobacterium demsielae*. *V. harveyi*, *Vibrio harveyi*. BuOH, *n*-butanol soluble extract.

DCM, dichloromethane soluble extract. EtOAc, ethyl acetate soluble extract. Hexane, *n*-hexane soluble extract. H_2_O, distilled water soluble extract.

**Table 3:** Antibacterial activity of erythromycin (ERY) and oxytetracycline (OTC) against bacterial strains.

Bacterial strain	Zone of inhibition (mm)	
	ERY (5 mg/disc)*	OTC (30 mg/disc)**
*Edwardsiella tarda* EET34	22.6 ± 0.5^d^***	9.6 ± 0.2^g^
*E. tarda* EET53	29.0 ± 0.3^b^	10.3 ± 0.5^f^
*E. tarda* EET54	29.0 ± 0.5^b^	12.6 ± 0.3^e^
*Photobacterium damselae* FP2137	29.3 ± 0.8^ab^	10.6 ± 0.4^f^
*P.damselae* FP2261	30.0 ± 0.5^a^	12.0 ± 0.5^e^
*P. damselae* FP4137	11.0 ± 0.5^g^	29.6 ± 0.5^c^
*Vibrio harveyi* RFHW9L	21.6 ± 0.4^e^	36.6 ± 0.4^b^
*V. harveyi* RFHW1KA	19.6 ± 0.3^f^	20.3 ± 0.4^d^
*V. harveyi* RFHW3KA	27.3 ± 0.2^c^	41.6 ± 0.3^a^

*Inhibition zone diameter interpretation: susceptible, ≥23; Intermediate, 14-22; and, resistant, ≤13 mm. Data are the averages of triplicate experiments.

**Inhibition zone diameter interpretation: susceptible, ≥15; Intermediate, 12-14; and, resistant, ≤11 mm. Data are the averages of triplicate experiments.

***Values sharing the same letters within each column are not significantly different at *p* < 0.05.

**Table 4:** Fractional inhibitory concentration (FIC) indices of erythromycin (ERY) in combination with EtOAc soluble fraction of *Eisenia bicyclis* methanolic extract against ERY-resistant fish pathogenic bacteria.

Bacterial strain	Tested compound	Antibiotic MIC	EtOAc fraction MIC	Combined MIC	FIC index	Interpretation
*E. tarda* EET34	ERY+EtOAc	64	128	32	0.75	SYN
*P. damselae* FP4137	ERY+EtOAc	256	128	64	0.75	SYN
*V. harveyi* RFHW9L	ERY+EtOAc	256	128	64	0.75	SYN
*V. harveyi* FRHW1KA	ERY+EtOAc	256	128	128	1.5	S-AD

*E. tarda*, *Edwardsiela tarda*. *P. damselae*, *Photobacterium demsielae*. *V. harveyi*, *Vibrio harveyi*. EtOAc, ethyl acetate. MIC, minimum inhibitory concentration (μg/ml). FIC index= MIC_combined_/MIC_alone_)+(MIC_combined_/MIC_EtOAc fraction_). Interpretation: SYN (synergistic), FIC index was <1. S-ADD (sub-additive), FIC index was between 1.0 and 2.0.

**Table 5:** Fractional inhibitory concentration (FIC) index of oxytetracycline (OTC) in combination with EtOAc soluble fraction of *Eisenia bicyclis* methanolic extract against OTC-resistant fish pathogenic bacteria.

Bacterial strain	Tested compound	Antibiotic MIC	EtOAc fraction MIC	Combined MIC	FIC index	Interpretation
*E. tarda* EET34	OTC+EtOAc	0.5	128	0.5	1.0	ADD
*E. tarda* EET53	OTC+EtOAC	512	128	512	>2.0	ANT
*E. tarda* EET54	OTC+EtOAC	512	128	1024	>2.0	ANT
*P. damselae* FP2137	OTC+EtOAC	256	256	1024	>2.0	ANT
*P. damselae* FP2261	OTC+EtOAC	512	256	1024	>2.0	ANT
*V. harveyi* FRHW1KA	OTC+EtOAC	64	128	128	>2.0	ANT

*E. tarda*, *Edwardsiela tarda*. *P. damselae*, *Photobacterium demsielae*. *V. harveyi*, *Vibrio harveyi*. EtOAc, ethyl acetate. MIC, minimum inhibitory concentration (μg/ml). FIC index= MIC_combined_/MIC_alone_)+(MIC_combined_/MIC_EtOAc fraction_). Interpretation: ADD (additive), FIC index was 1.0; ANT (antagonistic), FIC index >2.0.
